# Genomic alterations in the stepwise progression from normal mucosa to metastasizing oral squamous cell carcinoma

**DOI:** 10.3389/fonc.2024.1450361

**Published:** 2024-09-11

**Authors:** Jakob Myllerup Jensen, Sannia Mia Svenningsen Sjöstedt, Javiera Laing Carmona, Lise Barlebo Ahlborn, Filipe Garrett Vieira, Finn Cilius Nielsen, Katalin Kiss, Christian Grønhøj, Christian von Buchwald

**Affiliations:** ^1^ Department of Otorhinolaryngology, Head and Neck Surgery and Audiology, Rigshospitalet, University of Copenhagen, Copenhagen, Denmark; ^2^ Department of Clinical Physiology and Nuclear Medicine, Rigshospitalet, University of Copenhagen, Copenhagen, Denmark; ^3^ Department Clinical Physiology and Nuclear Medicine, Center for Functional and Diagnostic Imaging and Research, Copenhagen University Hospital, Hvidovre, Denmark; ^4^ Department of Genomic Medicine, Rigshospitalet, University of Copenhagen, Copenhagen, Denmark; ^5^ Department of Pathology, Rigshospitalet, University of Copenhagen, Copenhagen, Denmark

**Keywords:** oral squamous cell carcinoma, head and neck cancer, carcinoma, genomics, metastasis

## Abstract

**Introduction:**

The aim of this study was to investigate the genomic changes that occur in the development from dysplasia, cancer and to regional metastases in patients with oral cavity squamous cell carcinoma (OSCC).

**Material and methods:**

We included OSCC patients with lymph node metastases at diagnosis, treated with primary surgery at Rigshospitalet, University of Copenhagen in the period 2007-2014. The resected tumor specimens were evaluated by a pathologist, who marked areas of morphologically normal tissue and dysplasia surrounding the cancer, two areas from the cancer tissue, and one area within the lymph node metastases. From these areas a punch biopsy was taken, and DNA from each sample was extracted and sequenced using Illumina’s TSO500 HT cancer panel.

**Results:**

From 51 OSCC patients, 255 samples were included, comprising a wide variety of genomic alterations. Substantial intratumor heterogeneity was found. The most commonly mutated gene was *TP53*, mutated in 65% of all samples. Only two patients had no *TP53* mutation in any samples. We found that morphologically normal appearing mucosa as well as surrounding dysplasia also contained malignant mutations, supporting the theory of field cancerization. There was a significant lower average tumor mutational burden (TMB) in the lymph node metastases compared to the primary tumors, supporting the theory of clonal selection.

**Conclusion:**

Substantial inter- and intratumor genomic heterogeneity was found. Mutation of *TP53* was the most common and was present in all but two patients. Our data strongly supports the theory of clonal selection and the theory of field cancerization.

## 1 Introduction

Every year, nearly 400,000 patients are diagnosed with oral cavity squamous cell carcinoma (OSCC) worldwide (1). In Denmark, OSCC is the second most common head and neck cancer with an increasing incidence over the past decades, currently at 3.5 per 100,000 per year (2). OSCC is often associated with tobacco use and excessive alcohol consumption, which are also known to have a synergistic effect on the malignant development (3–5).

Studies have demonstrated that the genetic alterations in head and neck squamous cell carcinomas are heterogeneous without common mutational signatures (6–8) a finding which might be explained by the carcinogenic effect of tobacco and alcohol introducing random DNA alterations. Substantial intratumor genomic heterogeneity has previously been reported for head and neck cancer as well as squamous cell lung carcinoma, which also is frequently associated with tobacco smoke (6, 7, 9, 10).

OSCC often evolves in a stepwise progression from mucosal dysplasia to invasive carcinoma. However, not all cases of dysplasia undergo malignant transformation—in the cases where this occurs, dysplasia is often present adjacent to the malignant tissue (11). Metastatic spread of OSCC is most frequently to regional lymph nodes of the neck and, if feasible, surgery including cervical lymph node dissection is first-line treatment (12).

In this study, using paired tissue samples from surgically resected primary tumor and lymph node metastases from patients with OSCC, our aim was to investigate the genomic alterations that occur in the development from dysplasia to cancer, and further on to lymph node metastases. In doing so, identifying potential driver mutations which could provide us with a better understanding of the developmental process leading to OSCC.

## 2 Material and methods

OSCC patients with lymph node metastases at diagnosis treated with primary surgery were identified from the COrCa database, which is a consecutive, population-based database of 1399 OSCC patients treated at Rigshospitalet, University of Copenhagen in the period from 2007 to 2014 (13). Clinical data on the patients were obtained through the COrCa database. Patients with lichen planus were excluded.

### 2.1 Tissue samples

**Figure 1:**
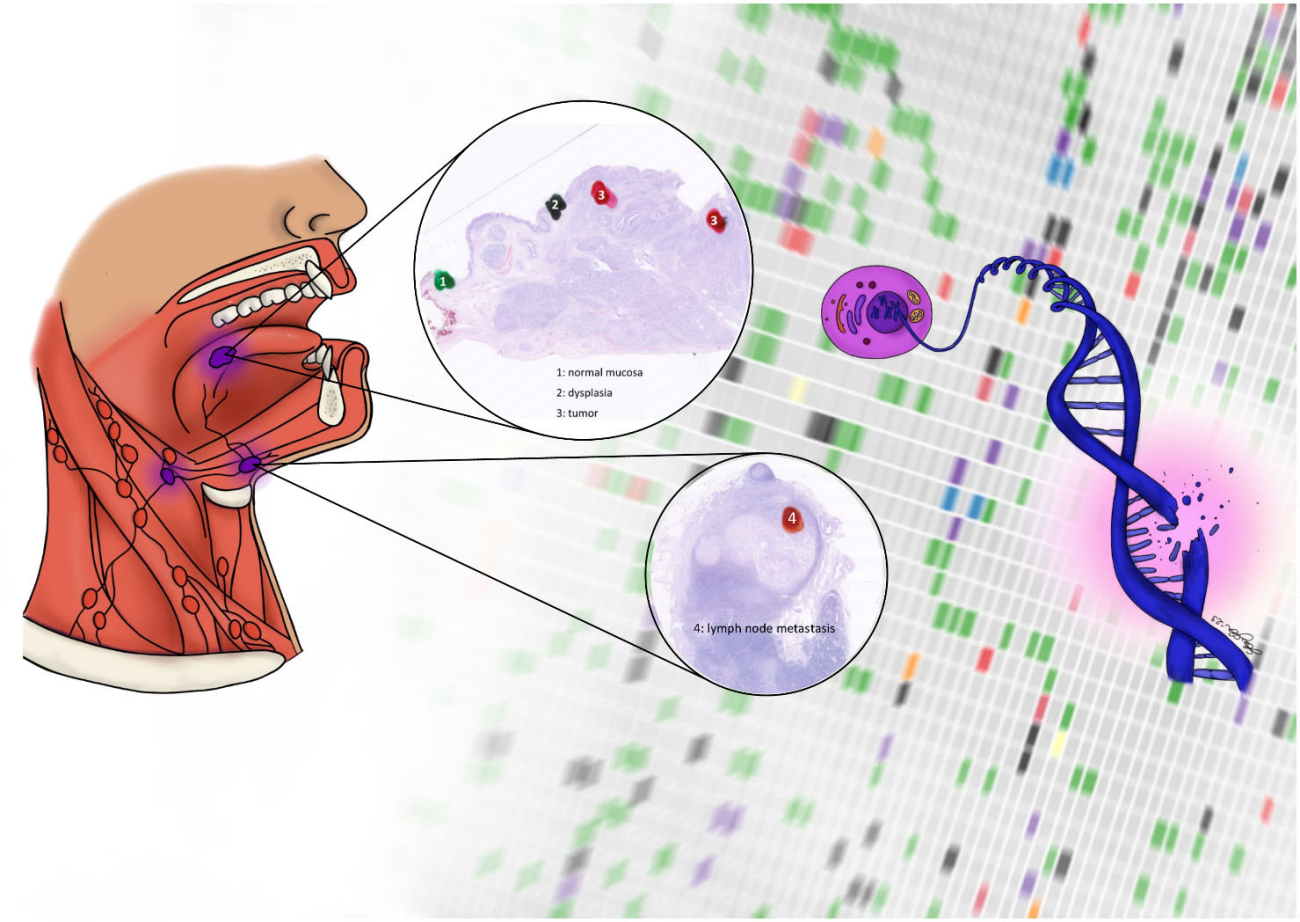
Surgical specimen containing morphologically normal tissue (1), dysplastic tissue (2), tumor (3), and lymph node metastasis (4)

Formalin-fixed paraffin embedded (FFPE) resected tumor specimens and lymph node metastases were identified from the local pathology archive at Rigshospitalet. Tumor specimens were handled according to standard operating procedures and evaluated by a pathologist, who marked areas containing 1) normal tissue in the periphery of the resected specimen, 2) dysplastic tissue surrounding the cancer, 3) two areas from the cancer tissue, and 4) lymph node metastases. From these areas a punch biopsy from the corresponding paraffin block was obtained (**Figure 1**). In the normal tissue, the punch biopsy was 2 mm in diameter, and from the remaining areas 1 mm in diameter. All biopsies were transferred to Eppendorf tubes.

### 2.2 Sequencing data generation and analysis

From each FFPE tumor specimen, DNA was extracted using GeneRead DNA FFPE Kit (Qiagen, Hilden, Germany) according to manufacturer’s instructions, except for twice the amount of proteinase K and deparaffination solution were used, and samples were left overnight for proteinase K digestion at 56°C. The libraries were prepared with Illumina’s TruSight Oncology 500 High-Throughput (TSO500 HT) cancer panel (containing 523 cancer-relevant genes) according to the manufacturer’s instructions, using between 113 - 6675 ng of DNA as input material depending on the available amount of DNA from the biopsies. The libraries were sequenced on an Illumina NovaSeq 6000 (2x150 basepairs) with a minimum average coverage >295x (range 295.3-1781.5). Raw sequencing data (.bcl files) were demultiplexed into individual FastQ read files with Illumina’s bcl2fastq v2.20.0 (Illumina Inc., San Diego, CA) based on their unique index, and each sequencing library quality checked with fastQC v0.11.8.

Sequenced reads were trimmed with BBduk v38.26, mapped to hg38/GRCh38 reference genome using BWA-MEM v0.7.15, and alignment quality control performed with mosdepth v0.2.6. Somatic variants were called for each tumor sample with GATK v4.1.9.0 suite’s Mutect2 using Best Practices guidelines for Tumor-only analyses (i.e. without a paired Normal sample) and an internal Panel-Of-Normals comprising 255 non-cancer samples and a minimum sample count (–min-sample-count) of 11. Furthermore, copy number alterations (CNAs) were called using local TSO500 app v. 2.2.0 as well as TMB estimation. For all relevant analyses, the germline resource used was generated from all gnomAD variant sites with a frequency greater than 3%.

### 2.3 Variant filtering

Filtering was performed using Qiagen Clinical Insight(QCI) software (Qiagen Bioinformatics, Aarhus, Denmark/Redwood City, CA, USA) including exclusion of common variants (variants more common than 0.5% in 1000 genomes project, ExAC, gnomAD, or NHLBI ESP exome), and a read depth of ≥10x. Only point mutations and CNAs that were classified as pathogenic or likely pathogenic using QCI software were included in the further data analysis and interpretation. Furthermore, CNAs were only reported if the fold change (fc) were above 2,2 (14).

### 2.4 Plots

For creation of the oncoplots indels longer than 10 bps, and variants with a coverage lower than 100x and a VAF lower than 5% were excluded. Further, all variants present in more than 20% of the samples were considered as germline or technical artifacts and therefore, also excluded from downstream analyses.

Oncoplots were generated with maftools R library (15). Signature plots were created using R package sigminer with hg38 reference genome package BSgenome.Hsapiens.UCSC.hg38 and Single base substitutions (SBS) mode (16, 17).

## 3 Results

In total, tissue samples from 51 patients with OSCC were included. In all patients, samples of morphologically normal oral mucosa, dysplastic oral mucosa, the primary tumor, and lymph node metastases were available, 255 samples in total. The median age at diagnosis was 63 years (IQR: 56-70), and the majority were men (n=35, 69%). Most patients were smokers or previous smokers at diagnosis (n=43, 84%), and 59% had an excessive alcohol intake or prior excessive alcohol intake (n=30). The tongue and the floor of the mouth (n=22, 43%, and n=21, 41%) were the most common sublocations, and most patients were diagnosed in UICC7 T-stage 2 (n=22, 43%) (**Table 1**).

**Table 1:** Baseline characteristics of included patients.

	N
Sex	
Male Female	35 (69%) 16 (31%)
**Median age at diagnosis [IQR]**	63 [56 – 70]
T-stage	
T1 (≤2cm) T2 (>2cm, ≤4 cm) T3 (>4cm) T4a/b/x (invading nearby structures) Unknown	12 (24%) 22 (43%) 7(14%) 5 (10%) 5 (10%)
Anatomical location	
Tongue Floor of mouth Other (mucosa buccalis, retromolar, gingiva)	22 (43%) 20 (39%) 9 (18%)
Smoking status	
Never smoker Former smoker Current smoker Unknown	6 (12%) 9 (18%) 34 (67%) 2 (4%)
Alcohol consumption	
Normal intake Prior excessive intake Excessive intake unknown	19 (37%) 8 (16%) 22 (43%) 2 (4%)

### 3.1 Genomic alterations

The number of point mutations and copy number alterations (CNAs) varied between patients. All patients harbored at least one pathogenic or likely pathogenic point mutation, however not all had relevant CNAs.

The highest number of point mutations in one patient was 16, while the highest number of CNAs in one patient was 13. The patients with highest number of genetic alterations in total had 21 different point mutations and CNAs.

Several of the genomic alterations present in the malignant tissue were also seen in the morphologically normal tissue samples of the same patients, and only six patients (12%) had no genomic alterations in their morphologically normal tissue sample. The remaining had at least one point mutation; no patients had only CNAs in their morphologically normal tissue sample.

Comparing matched tumor samples substantial intratumor heterogeneity was found. Only eight patients (16%) shared all genomic alterations in their matched tumor samples, and even in these cases the VAF and fold changes were different between the two samples.

### 3.2 Point mutations

**Figure 2:**
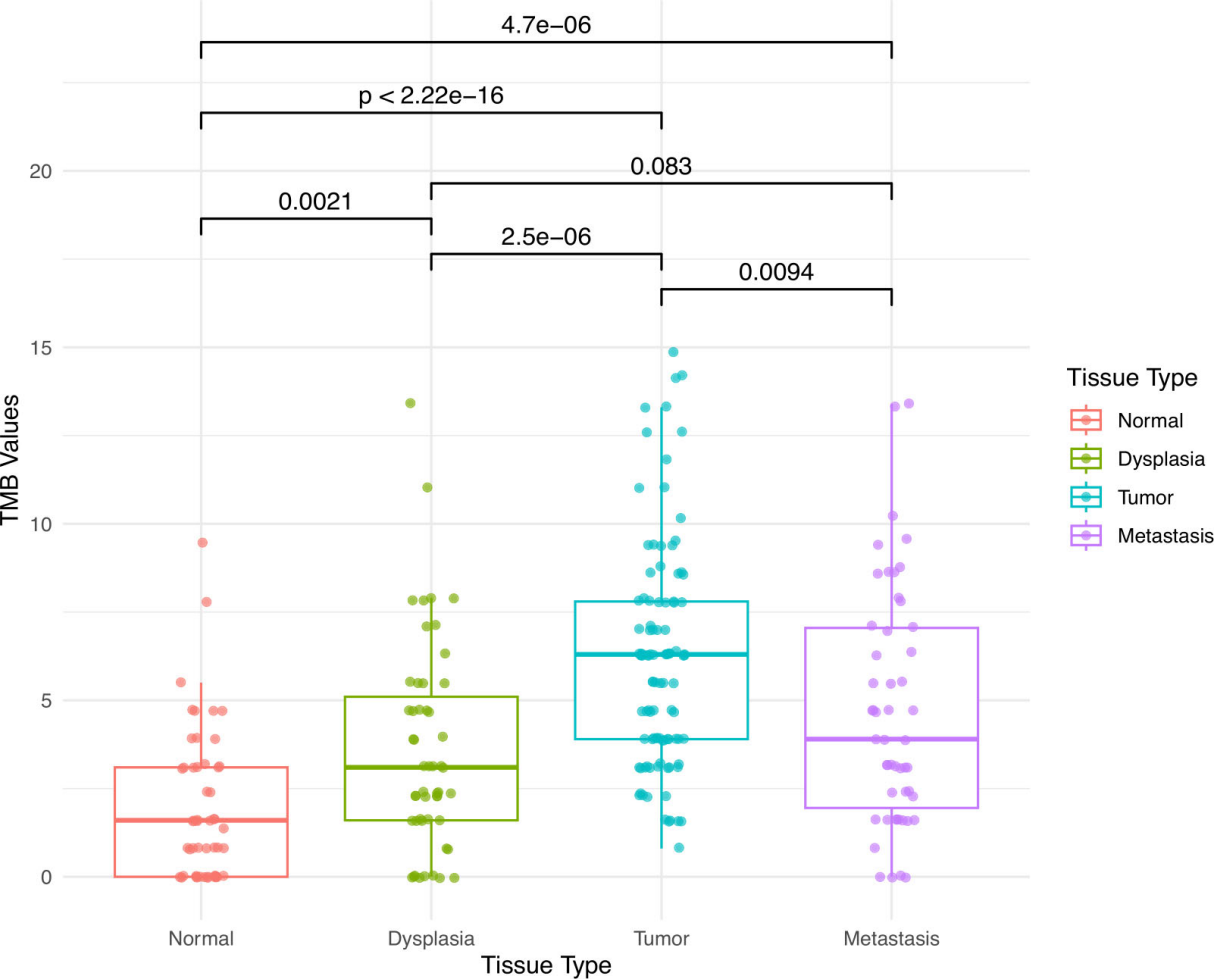
Tumor mutation burden (TMB) in morphologically normal mucosa, dysplasia, carcinoma, and lymph node metastases.

The overall average tumor mutational burden (TMB) score per sample was 4.5 mut/Mb. For the different tissue categories, the highest average TMB score was 6.1 mut/Mb for tumor samples, while normal tissue samples had the lowest average TMB score at 1.9 mut/Mb. Lymph node metastases had significantly lower average TMB score than tumor samples. There were significant differences in TMB score between all tissue categories, except between dysplasia and lymph node metastasis (**Figure 2**).

**Figure 3:**
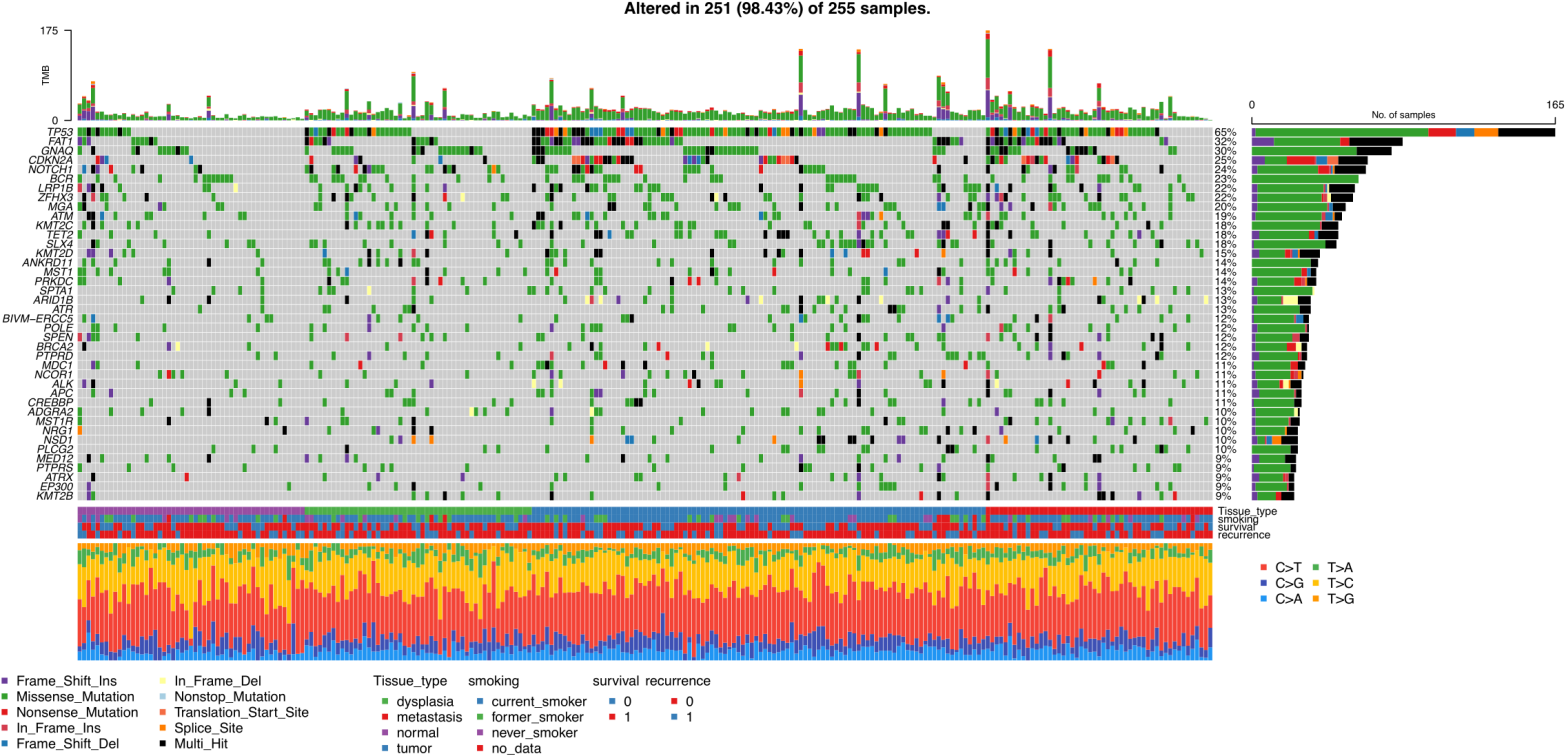
Oncoplot including all samples and 40 most frequently mutated genes, including information on type of mutation, and clinical data on patients.

Most variants were missense mutations, followed by frameshift and stop gain mutations. Alteration of *TP53* was the most frequent, altered in 65% of all samples. Other commonly mutated genes were *FAT1* (32%) and *CNAQ* (30%). Based on VAF of the most commonly mutated genes in the different tissue categories, *CDKN2A* appears to be a late event, occurring in the primary tumor, while *TP53*, *FAT1*, *QNAC*, *NOTCH1* and *BCR* are early events, arising in the morphologically normal tissue and dysplasia (**Table 2**, **Figure 3**).

**Table 2:** Frequency of the most common mutations during the various stages of OSCC development.

	Normal tissue N (%)	Dysplasia N (%)	Tumor N (%)	Lymph node metastasis N (%)
TP53	12 (23.5)	24 (47.1)	39 (76.5)	39 (76.5)
FAT1	9 (17.6)	12 (23.5)	34 (66.7)	20 (39.2)
GNAQ	11 (21.6)	19 (37.3)	29 (56.9)	17 (33.3)
CDKN2A	4 (7.8)	6 (11.8)	32 (60.7)	19 (37.3)
NOTCH1	8 (15.7)	18 (35.3)	24 (47.1)	11 (21.6)
BCR	12 (23.5)	12 (23.5)	24 (47.1)	11 (21.6)
LRP1	8 (15.7)	8 (15.7)	23 (45.1)	11 (21.6)

### 3.3 TP53

*TP53* mutations were very frequent; only two patients (4%) did not have any *TP53* mutations in any sample—the remaining patients had between one and seven different mutations in *TP53*. The most common variant, present in eight patients, was a well-known hotspot missense mutation (c.524G>A p.R175H). The normal tissue samples of 22 patients (41%) contained *TP53* mutations, in most cases in very low frequencies around 1-2%, and only in few cases the frequency increased noteworthy going through the pre-cancer, tumor, and metastasis tissue.

The patients (N=28, 55%) with no *TP53* mutations in their normal tissue who gained the mutation in the pre-cancer or tumor tissue often had higher frequencies and increasing frequencies throughout the development of the cancer.

### 3.4 Copy number alterations

Amplifications were seen in 31 different genes, the most common was *FGF3*, seen in 20 patients (39%), followed by *FGF19*, and *CCND1* seen in 18 (35%) and 26 (51%) patients, respectively. Opposite to the frequent presence of point mutations in normal tissue, amplifications were not seen in the normal tissue samples for any patient and did often not occur before tumor samples.

The highest fold change was seen for *FGF3* in a lymph node metastasis sample, at 16.4. *FGF3* had the highest fold change in dysplasia and tumor samples as well, 7.4 and 12.3, respectively. Other genes showing high fold change were *FGF19* (highest 14.4 in a lymph node metastasis sample), *CCND1* (highest 14.3 in a lymph node metastasis samples), and *EGFR* (highest 11,0 in a tumor sample).

### 3.5 Gene set enrichment during transition from normal epithelium to dysplasia and cancer

**Figure 4:**
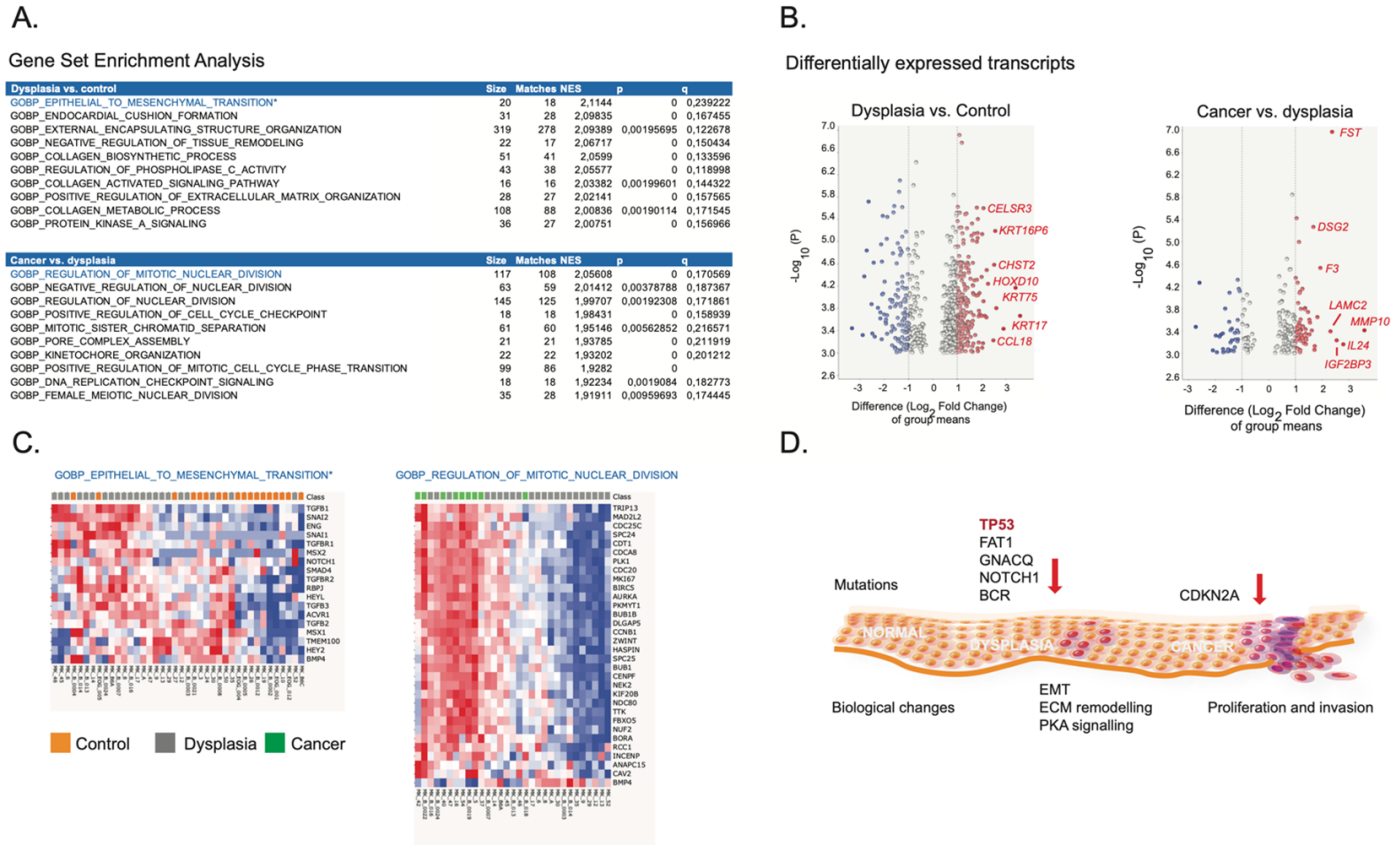
Biological processes during transition from normal cell to dysplasia and cancer in oral squamous cell carcinoma. **(A)** Gene set enrichment analysis (GSEA) of normal oral cells compared with cells undergoing dysplasia (upper panel) and of dysplastic cells compared with squamous cell carcinoma (lower panel). The normalized dataset was generated by Khan et al.21 and was downloaded from the gene expression omnibus (GEO) (GSE227919). The GSEA was performed as described20 in Qlucore Omics Explorer 3.9,0 www.qlucore.com employing the gene ontology biological processes C5 collection of 7647 gene sets available at https://www.gsea-msigdb.org/gsea/msigdb/human/collections.jsp#C5. The table panels show the 10 most enriched gene sets ranked according to their normalized enrichment score (NER). Moreover, the gene set size and matches, as well, the p and q values are indicated. **(B)** Volcano plot of transcripts exhibiting differential expression between normal cells and dysplasia (left panel) and dysplasia versus cancer (right panel). Upregulated transcripts are show to the right and are marked in red. Outstanding mRNAs are labelled with their gene name. **(C)** Two-way hierarchical clusters of the two in most enriched gene sets during the transition from normal cell to dysplasia and from dysplasia to cancer, respectively. The class is indicated below and shown on the top of the clusters. Sample codes from the study by Kahn et al. is shown below. **(D)** Schematic summary of the molecular alterations leading to dysplasia and cancer in oral squamous carcinoma. Mutations occurring during the transition from normal epithelium to dysplasia and cancer are indicated on the top of the schematic representation of the epithelium and the accompanying biological processes are shown below.

To characterize the biological processes during transition from normal epithelium to dysplasia and cancer in OSCC, we finally performed a gene set enrichment analysis employing gene expression data from Khan et al. (GSE227919), examining a cohort (n=66) of patients with early oral lesions consisting of premalignant lesions (hyperkeratosis and dysplasia) and OSCC as well as normal controls. We examined the gene ontology biological processes C5 collection as described (18). As shown in (**Figure 4**) there is an upregulation of gene sets involved in epithelium to mesenchymal transition (EMT) during progression to dysplasia, whereas, the progression from dysplasia to carcinoma mainly involves clonal expansion characterized by upregulation of check point and mitotic signaling (Panel B). This tendency is also apparent in the volcano plot where keratins are prominent in dysplasia and in the two-way clusters showing the clear mitotic profile in the carcinoma (C).

## 4 Discussion

Using targeted DNA sequencing, we analyzed 255 paired tissue samples from 51 patients with OSCC. From each patient samples were obtained from morphologically normal mucosa, mucosal dysplasia, two samples from the primary cancer, and lymph node metastases. In line with previous studies, the mutational landscape of OSCC tumors as well as dysplasia and lymph node metastases was heterogeneous, with OSCC tumors also demonstrating substantial intratumor heterogeneity (6, 7, 10, 19). Several of the mutations found in the dysplasia and primary tumor samples were also found in the surrounding morphologically normal tissue, supporting the theory of field cancerization, in which the long-term exposure to carcinogens such as tobacco induces potentially carcinogenic genomic alterations in cells throughout the exposed mucosa (11, 20, 21). - leaving the mucosa which has not undergone malignant transformation in increased risk of doing so over time owing to the accumulation of genomic alterations, which clinically manifests as multifocal carcinoma and/or a tendency to mucosal recurrence outside of the initial T-site (22).

Our data indicate that mutation of *TP53*, as well as *FAT1, GNAQ, NOTCH1*, and *BCR* seems to be early events, arising in the morphologically normal tissue and dysplasia. We found *TP53* to be the most frequently mutated gene identified in 49 patients (96%). *TP53* is a well-known tumor suppressor gene associated with many different types of cancer, and has been reported as the most frequently mutated gene in OSCC (23–30). *TP53* encodes p53 that plays an important role in activating DNA repair and arresting cell growth, as well as inducing apoptosis in cells with DNA damage. Mutation of *TP53* has been linked with the multistep process of EMT, including acquiring the ability to break down extracellular matrix, tumor invasion, as well as intra- and extravasation (31, 32). In line with this, mutation of *TP53* has been shown to be associated with increased migratory and invasive potential and may increase risk of malignant transformation to OSCC when found in dysplasia (25, 32).

Like *TP53*, *NOTCH1*, and *FAT1*, are involved in EMT through the NOTCH/Jagged pathway and FAT1/HIPPO pathway, respectively (33–35). Knockdown of *GNAQ* has been shown to induce mesenchymal stem cell like properties in lung cancer cells (36, 37).

Mutation of *CDKN2A* appears to be a late event, occurring in the primary tumor. *CDKN2A* affects cell cycle regulation, and encodes both p16(INK4a), which is an inhibitor of cyclin dependent kinase (CDK) and p14(ARF), a p53 stabilizer (38, 39). Germline mutation of *CDKN2A* has been linked with early onset OSCC (38).

In this way, the variant data implies that EMT in the dysplastic foci precedes clonal expansion of malignant cells in the tumor. The general view is in line with the gene expression data from Khan et al (19), where a gene set enrichment analysis (GSEA) showed that premalignant lesions were enriched in gene signatures associated with cellular plasticity with partial EMT phenotypes, and immune response. Our stepwise analysis of data from Khan et al. indicates that going from normal tissue to dysplasia is characterized by EMT and that tumors exhibit a strong mitotic component compared to the dysplastic foci.

Previous studies show increased risk of malignant transformation over time in benign oral lesions with higher grades of dysplasia, but it has also been suggested that underlying genetic alterations do not necessarily correlate with histomorphology, as some lesions with mild dysplasia have demonstrated genetic alterations similar to those found in lesions with severe dysplasia (40, 41). Patterns of immune infiltration of OSCC may also be linked with prognosis in OSCC (42) – to the best of our knowledge, definitive biomarkers to distinguish between lesions that remain benign and lesions that will undergo malignant transformation has yet to be discovered.

In head and neck squamous cell carcinoma (HNSCC), amplification of 11q13, containing among others *CCDN1, FGF3*, and *FGF19*, is frequent and has been linked with a poor prognosis (43, 44). The most frequent amplification in our study was *FGF3*, seen in 20 patients (39%). This has also been reported as a frequent amplification in OSCC by Nakagaki et al. and Ribeiro et al., along with amplification of *CCND1*, which was also among the most frequent in our study—co-amplification of *FGF3* and *CCND1* was often observed in their cohort as well as in our cohort (28, 45). *CCND1* is also frequently co-amplified with *FGF19* in head and neck squamous cell carcinoma, amplification of which was seen in 18 (35%) of patients in our cohort—*FGF19* is involved in HNSCC tumorigenesis and may be useful as a target for therapy (46).

From a clinical perspective, the results of this study, with clear support of the theory of field cancerization, might explain the relatively frequent local recurrences seen in OSCC, even among patients treated radically with surgery. It also underlines the importance of smoking cessation after diagnosis of OSCC. With continued smoking follows a high risk of inducing further malignant mutations in already exposed mucosa.

Unfortunately, our results also indicates that there are no obvious common mutational patterns that can serve as a target of therapy to be used in the broad group of OSCC patients. Targeted therapy probably needs to be individualized to each specific patient, based on their specific mutations.

The strengths of this study lie in the large number of patients, with matched tissue samples from morphologically normal mucosa, dysplasia, tumor, and lymph node metastasis as well as the availability of clinical data. As less than 20% of patients with oral dysplasia will progress to OSCC, future studies should explore differences in dysplasia in patients that later develop OSCC and patients that do not develop OSCC to identify potential biomarkers of increased risk of malignant transformation.

## 5 Conclusion

Based on targeted DNA analysis of 255 paired samples from 51 OSCC patients, we found substantial inter- and intratumor genomic heterogeneity. Mutation of the well-known cancer gene *TP53* was present in all but two patients. We found that morphologically normal appearing mucosa as well as surrounding dysplasia also contained mutations, supporting the theory of field cancerization and mucosa at risk. Our data as well as data from a previous study by Khan et al. indicates that the development from normal tissue to dysplasia is characterized by EMT, and the development from dysplasia to cancer are characterized by mutations in regulators of proliferation.

## Data Availability

According to Danish data protection law, data from this study are not allowed to be made publicly available. Transfer of data requires permission from the Danish data protection authorities for the specific transfer. This can be applied for upon request to the corresponding author.
